# Emergent Role of Intra-Tumor Radioactive Implantation in Pancreatic Cancer

**DOI:** 10.3390/cancers18020302

**Published:** 2026-01-19

**Authors:** Pathipat Durongpongkasem, Amanda H. Lim, Nam Q. Nguyen

**Affiliations:** 1 Department of Gastroenterology and Hepatology, Royal Adelaide Hospital, Adelaide, SA 5000, Australia; 2 Discipline of Medicine, The University of Adelaide, Adelaide, SA 5000, Australia

**Keywords:** pancreatic cancer, phosphorus-32 microparticles, intra-tumoral radiation, chemotherapy

## Abstract

Pancreatic ductal adenocarcinoma (PDAC) remains one of the most lethal malignancies, with limited treatment options for patients with locally advanced or metastatic disease. Endoscopic ultrasound (EUS)-guided intra-tumoral radioactive implantation has emerged as a minimally invasive approach to enhance local tumor control while minimizing systemic toxicity. Among the available isotopes, phosphorus-32 (^32^P) microparticle brachytherapy has demonstrated promising outcomes, including significant tumor regression, reductions in CA 19-9, and higher rates of tumor downstaging and surgical conversion when combined with systemic chemotherapy. Compared with stereotactic body radiotherapy (SBRT), ^32^P delivers higher intratumoral radiation doses, spares adjacent healthy tissues, and can be administered during ongoing chemotherapy without treatment interruption. Additionally, preliminary evidence suggests that ^32^P may modulate the tumor microenvironment, improving vascularity and enhancing chemotherapy efficacy. The procedure shows high technical success and a favorable safety profile, with minimal serious adverse events. Future directions include prospective randomized trials to validate its impact on survival, optimize dosing, and establish treatment protocols. EUS-guided intra-tumoral ^32^P brachytherapy holds potential as a key component of multimodal therapy, bridging local tumor control and systemic disease management in PDAC.

## Simple Summary

Pancreatic ductal adenocarcinoma remains a silent killer and is in dire need of more effect therapeutic strategies. Endoscopic ultrasound (EUS) has expanded its repertoire over the last two decades and can be used as a mode of treatment delivery directly into pancreatic tumors. In this review, we provide details on EUS-guided intra-tumoral fine-needle injectional therapies, highlighting the use of intra-tumoral radioactive implantation in patients with locally advanced pancreatic cancer.

## 1. Introduction

Pancreatic cancer remains one of the deadliest malignancies worldwide, ranking as the fifth leading cause of cancer-related death globally and the third most common cause in both the USA and Australia [1,2]. According to GLOBOCAN 2018, the incidence and mortality of pancreatic cancer are projected to increase dramatically—by 77.7% and 79.9%, respectively—between 2018 and 2040 [3]. The persistently high mortality is driven by aggressive local invasion and early systemic dissemination. Surgical resection is the only potentially curative option, yet fewer than 20% of patients are eligible at diagnosis. The majority present with unresectable locally advanced or metastatic disease [4].

Current standard therapy for these patients relies on systemic chemotherapy, typically FOLFIRINOX (leucovorin, fluorouracil, irinotecan, and oxaliplatin) or gemcitabine plus nab-paclitaxel in patients with adequate performance status, and gemcitabine alone for those unable to tolerate more intensive regimens, sometimes combined with radiotherapy [4,5,6]. Despite these treatments, outcomes remain poor, with an median overall survival of 18–24 months for locally advanced pancreatic cancer (LAPC) and 8–11 months for metastatic disease [7]. Treatment efficacy is further limited by intrinsic and acquired chemoresistance [8], highlighting the need for novel therapeutic approaches.

In recent years, intra-tumor brachytherapy has emerged as a promising intervention aimed at improving local tumor control, prolonging survival, and minimizing systemic toxicity. This review will examine the current evidence and evolving role of intra-tumoral radiation therapy in the management of pancreatic cancer.

## 2. Tumor Barriers and Chemoresistance in Pancreatic Cancer

Patients with advanced pancreatic ductal adenocarcinoma (PDAC) continue to face poor survival outcomes, largely due to low response rates to therapy, frequent tumor relapse, and early metastasis. Understanding the mechanisms underlying PDAC progression and chemoresistance is therefore critical for developing more effective treatment strategies.

A major contributor to therapeutic failure in PDAC is its unique tumor microenvironment (TME), which encompasses the surrounding extracellular matrix, stromal cells, and vasculature [9]. The PDAC TME is characterized by dense fibrosis, hypoxia, and immunosuppression, all of which impede drug delivery and attenuate anti-tumor immune responses. Key cellular components, including pancreatic stellate cells (PSCs) and cancer-associated fibroblasts (CAFs), secrete excessive extracellular matrix (ECM) proteins, generating tissue stiffness and creating physical barriers that hinder chemotherapy penetration. Signaling pathways such as Sonic Hedgehog (Shh) and transforming growth factor-beta (TGF-β) further activate PSCs and CAFs, driving fibrosis and tumor progression [10,11].

A defining feature of PDAC is its pronounced desmoplastic reaction, which, along with elevated interstitial fluid pressure from hyaluronan and proteoglycan accumulation, disrupts tissue architecture and limits therapeutic access. This dense stroma also contributes to hypovascularity, reducing perfusion and drug delivery. Hypoxia and desmoplasia interact in a feed-forward loop, exacerbating fibrosis, promoting tumor growth, and enabling immune evasion [12]. Although systemic therapies targeting the stroma—such as Shh inhibitors, TGF-β inhibitors, and hyaluronidase—have been explored, clinical outcomes have been largely disappointing, with minimal impact on survival [13,14].

These challenges highlight the need for strategies that bypass systemic delivery limitations. Direct intra-tumoral therapies, including local radiation and ablation, offer a compelling approach by achieving high local drug or radiation concentrations, enhancing tumor control, and potentially overcoming chemoresistance imposed by the dense PDAC stroma. This rationale underpins the growing interest in intra-tumoral brachytherapy and other locoregional interventions in pancreatic cancer.

## 3. Concept of Direct Tumoral Injectional Therapy

Direct intra-tumoral therapy represents a promising strategy to bypass the physical and vascular barriers that limit drug delivery in pancreatic cancer. By delivering therapeutic agents directly into the tumor, this approach achieves high local concentrations while minimizing systemic exposure and toxicity. Beyond enhanced cytotoxicity, direct injection also offers the potential to modulate the immunosuppressive tumor microenvironment, disrupting the stromal barriers that promote tumor progression and chemoresistance.

While percutaneous injections under ultrasound or CT guidance have demonstrated feasibility and safety, these approaches are technically challenging and cumbersome, particularly when repeated treatments are required [15]. Endoscopic ultrasound (EUS) has emerged as a transformative tool in this context. Originally a diagnostic modality, EUS now provides high-resolution, real-time imaging of pancreatic masses and surrounding structures, enabling precise, minimally invasive delivery of intra-tumoral therapies [16].

EUS-guided fine-needle injection (EUS-FNI) has been successfully applied to a variety of therapeutic strategies, including chemotherapy, immunotherapy, gene therapy, and ablative techniques such as radiofrequency ablation, laser therapy, cryotherapy, and ethanol injection. Additionally, EUS allows for the implantation of fiducial markers for stereotactic body radiation therapy (SBRT) or radioactive seeds for brachytherapy, further integrating diagnostic and therapeutic interventions in a single session.

Clinical studies have demonstrated that EUS-guided intra-tumoral therapy is both technically feasible and safe, with low rates of procedure-related complications (Table 1). A single prospective study has been published with regard to EUS-guided chemotherapy (gemcitabine) in LAPC, demonstrating only modest results [17]. Ablative therapies in LAPC includes EUS-guided radiofrequency ablation (RFA), which is a minimally invasive thermal therapy, reported for not only LAPC, but also for neuroendocrine tumors and pancreatic cystic neoplasms [18,19,20,21,22,23,24,25]. Although its feasibility and technical success have been shown to be positive, and although it has good safety profile, the benefits have not been overwhelmingly positive. Several studies have assessed EUS-guided gene therapy and immune-induced local therapy, including oncolytic viruses; however, these have not become part of clinical practice [18,20,26,27,28,29,30,31,32,33]. This is because the results have been variable, the study sample sizes included have been small, the backbone standard-of-care therapy used has not been current, and, in a single comparative trial (>10 years ago), there was no benefit compared to standard therapy. Robust data from prospective trials and randomized controlled studies confirming survival benefit are required. Nonetheless, the precision, versatility, and minimally invasive nature of EUS-guided therapy position it as a paradigm-shifting approach in the management of locally advanced pancreatic cancer, bridging the gap between systemic therapy and definitive local tumor control.

## 4. Radiotherapy in Pancreatic Adenocarcinoma

Radiotherapy plays an evolving role in the management of pancreatic ductal adenocarcinoma (PDAC), particularly as part of multimodal treatment strategies. Beyond direct cytotoxicity, radiation exerts complex effects on the tumor microenvironment (TME), including both immunostimulatory and immunosuppressive changes. By inducing immunogenic cell death, radiotherapy promotes the release of tumor-associated antigens, enhances antigen presentation, and stimulates effector T-cell infiltration, potentially converting the TME from an immunologically “cold” state into a “hot” state [34].

In resectable and borderline resectable PDAC, neoadjuvant chemoradiotherapy has been shown to improve surgical outcomes, including higher rates of R0 resection, improved local tumor control, and some evidence of survival benefit [35,36,37]. In locally advanced or unresectable disease, radiotherapy—particularly stereotactic body radiotherapy (SBRT)—is increasingly used to achieve local control, relieve symptoms, and occasionally convert tumors into resectable status [38]. Meta-analyses report surgical conversion rates of 7–21% following SBRT, with selected patients achieving margin-negative (R0) resections [39,40].

SBRT delivers highly focused, ablative doses over a limited number of fractions (typically 3–5), maximizing the sparing of adjacent critical structures while reducing overall treatment time compared with conventionally fractionated radiation therapy (CFRT). In locally advanced PDAC, SBRT has been associated with improved 2-year overall survival compared with CFRT (26.9% vs. 13.7%) and substantially lower rates of severe acute toxicity (5.6% vs. 37.7%) [41]. Accurate targeting with SBRT requires fiducial markers for motion tracking and CT-based treatment planning. Endoscopic ultrasound (EUS)-guided placement of fiducials is preferred due to the minimally invasive nature of this approach and its ability to access lesions throughout the pancreas, including those inaccessible percutaneously or surgically. Technical success rates exceed 90%, with minimal risks of complications or marker migration [42,43,44].

Despite its advantages, SBRT can disrupt systemic therapy schedules and exerts mixed effects on the TME. While it induces immunogenic tumor cell death, SBRT may also promote collagen deposition and stromal fibrosis without substantially reducing immunosuppressive cell populations, potentially limiting the efficacy of immune-based therapies [45].

In this context, direct intra-tumoral brachytherapy has emerged as a promising alternative, allowing selective escalation of radiation doses within the tumor while being deliverable concurrently with systemic therapy, circumventing many of the logistical and biological limitations associated with conventional SBRT.

## 5. EUS-Guided Intra-Tumoral Brachytherapy: A Targeted Approach

While SBRT provides focused, ablative radiation, its reliance on external beam delivery and fiducial markers can interrupt systemic therapy schedules and is limited by tumor location, surrounding critical structures, and stromal barriers. Direct intra-tumoral brachytherapy offers a complementary strategy by delivering high-dose radiation precisely within the tumor, maximizing cytotoxicity while sparing adjacent normal tissues.

Endoscopic ultrasound (EUS) has enabled minimally invasive access for brachytherapy, allowing the placement of radioactive seeds directly into pancreatic tumors under real-time imaging guidance. This approach combines the advantages of localized dose escalation with the ability to continue systemic chemotherapy uninterrupted, addressing two major limitations of SBRT. Additionally, EUS-guided brachytherapy provides the flexibility to treat lesions in anatomically challenging locations, including the pancreatic head, the uncinate process, or deep-seated tumors not amenable to percutaneous access.

**Figure 1:**
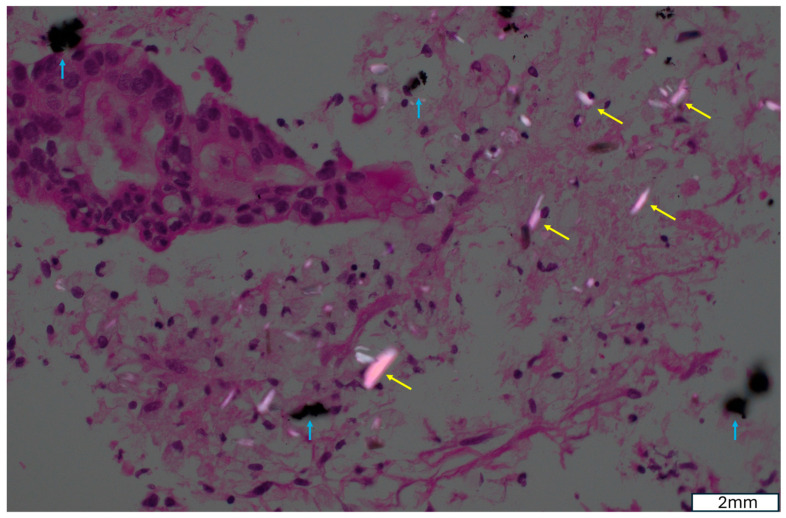
Polarizing light microscopy demonstrating bright needle-shape crystals (yellow arrows) accompanying black ^32^P particles (blue arrows) within the fibrotic tumor.

Early clinical studies suggest that EUS-guided intra-tumoral brachytherapy is technically feasible and safe, with low rates of procedure-related complications. By precisely delivering sustained radiation to the cancer only, this method can not only improve tumor control, enhance radiosensitivity within the dense desmoplastic stroma of pancreatic tumors, and potentially be synergized with systemic therapies and immunomodulatory strategies, but it can also improve safety profiles. The targeted intra-tumoral implantation of the radioactive isotope minimizes the risk of radiation-induced injury to the normal pancreatic tissue. With a short distance of radiation, intra-tumoral implantation of iodine-125 (^125^I) seeds and phosphorus-32 (^32^P) micro-particles has not been associated with pancreatitis. In addition, unlike the old colloid-based ^32^P particles [46], the new ^32^P micro-particles (known as Oncosil) are micro-crystals (Figure 1), which prevent the migration of ^32^P micro-particles outside of the cancer, thus minimizing the risk of radiation-induced injury to the normal pancreatic parenchymal or surrounding organ.

As such, EUS-guided brachytherapy represents a promising evolution in the multimodal management of locally advanced pancreatic cancer, offering a precision-based, minimally invasive alternative to conventional external beam radiotherapy. Additional advantages of EUS-guided intra-tumor brachytherapy include precise positioning, continuous real-time observation during implantation, and shorter puncture distance when compared to CT-guided percutaneous implantation [47,48]. The intra-tumor brachytherapy in LAPC has been studied with two main isotopes, which are ^125^I seeds and ^32^P micro-particles. Whilst other brachytherapy options have been investigated such as iodine-130 and yttrium-90, these studies are in the very early stages, and these options have yet to be investigated in human clinical trials.

### 5.1. ^125^I Radioactive Brachytherapy in Pancreatic Cancer

^125^I is a radioactive isotope of iodine commonly implanted as small solid seeds capable of emitting gamma radiation with a total dose of 140–160 Gy and a long half-life of approximately 60 days [49]. While ^125^I offers the advantages of sustained, localized radiation delivery, studies to date have not demonstrated a clear survival benefit in pancreatic cancer.

Current clinical trials of EUS-guided ^125^I brachytherapy have focused on patients with unresectable pancreatic cancer, including stage III and IV disease. These studies consistently demonstrate technical feasibility, minimal serious complications, and transient pain relief. However, median overall survival (OS) following implantation remains limited, ranging from 9 to 10.6 months [50,51,52,53]. A meta-analysis further confirmed that median OS with EUS-guided ^125^I seed implantation is generally under 12 months, comparable to outcomes achieved with chemotherapy alone or combined chemoradiation in similar patient populations [54,55].

Despite its minimally invasive nature, ^125^I brachytherapy presents several procedural challenges. Implantation requires multiple interstitial punctures—up to 30 seeds per session—using a 19-gauge needle. The stiffness of the needle can compromise placement accuracy, increasing the risk of post-procedural pancreatitis. Moreover, achieving uniform seed distribution is technically demanding, even with treatment planning system (TPS) guidance. Inadequate distribution can create “cold spots,” where subtherapeutic radiation doses reduce treatment efficacy. Notably, patients receiving a peripheral radiation dose exceeding 90 Gy demonstrated a partial remission rate of 80% (12/15 patients), whereas those receiving lower doses experienced poor tumor response [52].

While EUS-guided ^125^I implantation remains a promising minimally invasive option for localized radiation delivery in unresectable pancreatic cancer, further optimization of implantation techniques, dosimetry, and combination strategies is needed to improve clinical outcomes.

### 5.2. ^32^P Microparticle Brachytherapy in Pancreatic Cancer

Recent studies investigating ^32^P microparticles in patients with locally advanced pancreatic cancer (LAPC) and metastatic disease have shown promising outcomes. ^32^P is a radioactive isotope contained within silicon microparticles that emits pure beta radiation, delivering 98% of its dose within 81 days, with a half-life of 16 days. This allows localized radiation doses ranging from 100 Gy to 400 Gy directly to the tumor [56].

An initial cohort study of LAPC patients receiving ^32^P implantation alongside systemic chemotherapy (FOLFIRINOX or gemcitabine plus nab-paclitaxel) demonstrated a 90% local control rate at 16 weeks, with a mean tumor volume reduction of 26.4% and CA 19-9 decline of 34%. Importantly, tumor downstaging enabled surgical resection in 23.8% of patients initially deemed inoperable, with R0 resection achieved in 80% of these cases [57]. Similarly, a retrospective comparison between combination therapy (^32^P plus chemotherapy) and chemotherapy alone revealed that the addition of ^32^P doubled the tumor downstaging rate (31.4% vs. 13.6%), achieved greater tumor shrinkage at six months (−15.9 mm, *p* < 0.001), and increased surgical resection rates (28.6% vs. 12.1%). Moreover, combination therapy was associated with a longer restricted mean survival time (RMST) within 30 months post-chemotherapy (189 days) and improved local progression-free RMST (168.6 days) [58].

**Figure 2:**
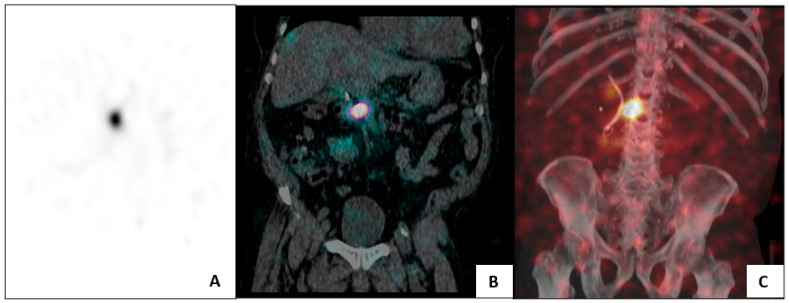
Bremsstrahlung imaging (**A**) and SPECT-CT scan (**B**,**C**) in patient after Oncosil implantation within 4 h to confirm localization and retention of a β-emitting isotope within the pancreas. These images from our cohort demonstrate that the ^32^P isotope has remained in the head of the pancreas with no ectopic distribution.

Further evidence supports the advantage of EUS-guided ^32^P implantation over stereotactic body radiotherapy (SBRT). In a retrospective cohort of LAPC patients, combination chemotherapy with EUS-guided ^32^P led to greater reductions in tumor size and CA 19-9 levels at six months, higher rates of tumor downstaging, more frequent surgical resections, and longer median overall survival (HR 0.45; *p* = 0.014) compared with chemotherapy plus SBRT [59]. Several factors likely contributed to these favorable outcomes. First, ^32^P delivers higher cumulative radiation doses (≈100 Gy) compared with the maximum achievable with external beam radiotherapy (EBRT) or SBRT (typically 40–50 Gy). Its beta radiation has limited tissue penetration (<1 cm), permitting higher localized doses while minimizing collateral exposure. Bremsstrahlung imaging or SPECT-CT within four hours post-implantation allows verification of localized distribution and exclusion of off-target radiation (Figure 2).

**Figure 3:**
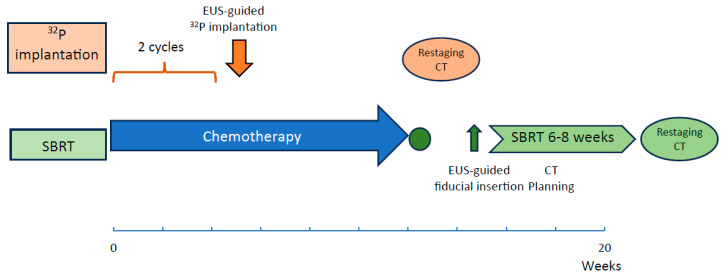
Diagram comparing the timeline between ^32^P implantation and SBRT. ^32^P implantation is performed after two cycles of chemotherapy as a single procedure, requiring a restaging CT at 3–4 months post-commencement of chemotherapy. In the case of SBRT, it is performed after a long period of chemotherapy, and requires interruption of chemotherapy for at least 6–8 weeks.

Importantly, early implantation of ^32^P during initial chemotherapy cycles may produce synergistic effects with systemic therapy, unlike SBRT, which is usually administered after completing chemotherapy and requires fiducial marker placement and CT planning (Table 2). Early ^32^P implantation also avoids delays in restaging or surgery inherent to SBRT logistics (Figure 3). Additionally, ^32^P may overcome pancreatic tumor microenvironment barriers. Lim et al. reported increased microvascular flow at weeks 4 and 12 post-implantation on CE-EUS imaging, suggesting improved vascularity and the potential enhancement of chemotherapy delivery [60]. Treatment can typically resume chemotherapy shortly after implantation, with tumor response assessed at 4–12 weeks (Figure 3).

Technical success rates for EUS-guided ^32^P implantation are consistently reported to be 100% in pilot and multicenter studies [57,58,61]. Safety data are favorable, with rare serious procedure-related adverse events. Minor side effects, such as transient abdominal pain or low-grade fever, are infrequent and self-limited, while grade ≥3 events attributable to the procedure are uncommon (<10%) [57].

In patients with metastatic PDAC, ^32^P implantation combined with chemotherapy has demonstrated 100% local disease control at three months, significant reduction in primary tumor diameter (−25%, *p* = 0.008), and improved quality of life at 12 weeks, highlighting its potential benefit in palliative settings [62].

Overall, EUS-guided ^32^P microparticle brachytherapy offers a unique combination of high local efficacy, safety, and potential synergistic effects with systemic therapy, supporting its role as a promising adjunctive local treatment for pancreatic ductal adenocarcinoma.

### 5.3. Other Radioactive Agents for Brachytherapy in Pancreatic Cancer

Several alternative radioisotopes have been explored for intra-tumoral brachytherapy in pancreatic ductal adenocarcinoma (PDAC), each with distinct physical properties and potential therapeutic benefits (Table 3).

#### 5.3.1. Holmium-166 (^166^Ho) Poly-L-Lactic Acid Microspheres (^166^HoMS)

^166^HoMS is an emerging locoregional therapy for PDAC delivered via intratumoral injection. With a short physical half-life of 26.8 h (compared with 14.3 days for ^32^P), ^166^Ho delivers a high dose rate over a brief period. Its β^−^ emission has limited tissue penetration, producing a steep dose fall-off that spares surrounding organs and major vasculature, as demonstrated in preclinical studies [63]. Furthermore, a unique advantage of ^166^HoMS is multimodal imaging capability, enabling SPECT, CT, and MRI for precise guidance and quantitative dosimetric evaluation. The first in-human feasibility study in unresectable PDAC showed that intratumoral ^166^HoMS injection is technically achievable and appears safe, though only three patients have been treated, and no survival benefit or efficacy data are yet available [64]. Further research is needed before routine clinical application.

#### 5.3.2. Palladium-103 (^103^Pd)

^103^Pd has been investigated primarily as an intraoperative interstitial implant for unresectable pancreatic cancer. Its low-photon gamma-ray energy, rapid dose fall-off, and short half-life may allow for higher localized dose rates with improved sparing of adjacent tissue compared to ^125^I. Two small clinical studies including 26 patients demonstrated feasibility and the palliation of symptoms, particularly pain, when ^103^Pd brachytherapy was combined with external beam radiation and chemotherapy. However, median survival remained limited (6.9–10 months), and high matched peripheral doses (>115 Gy) were associated with significant complications, including duodenal perforation, sepsis, cerebrovascular events, and radiation enteritis [65,66].

#### 5.3.3. Yttrium-90 (^90^Y)

^90^Y is a pure β-emitter with moderate tissue penetration. Compared with ^32^P, ^90^Y has a much shorter half-life (64.5 h vs. 343.2 h), delivering approximately 87% of its total radiation energy within 7 days, whereas ^32^P requires roughly 42 days [56,67]. Clinically, ^90^Y is primarily utilized for transarterial radioembolization (TARE) in hepatic malignancies, including liver metastases from pancreatic cancer. Retrospective studies suggest that ^90^Y TARE can achieve disease control and modest survival benefits (median OS 7–9 months) in patients with liver-dominant metastatic PDAC who have progressed on systemic therapy, with favorable toxicity profiles [68,69,70]. Improved hepatic progression-free survival has been observed particularly among patients aged < 65 years and those exhibiting stable or decreased CA 19-9 levels following treatment [68]. Early experimental work in porcine models has demonstrated that EUS-guided intratumoral implantation of ^90^Y microspheres is technically feasible and safe, producing necrotic lesion volumes between 255.8 and 745.6 mm^3^ using a 22-gauge needle [71]. Clinical studies evaluating EUS-guided ^90^Y brachytherapy in unresectable pancreatic cancer patients are currently underway.

#### 5.3.4. Iridium-192 (^192^Ir)

^192^Ir, unlike many of the other isotopes discussed, is a high-dose-rate brachytherapy and has been shown to be beneficial, particularly/for pain relief in patients with unresectable tumors undergoing bypass surgery to prevent jaundice [72]. Further assessment is required before deciding whether there is a role for ^192^Ir in pancreatic cancer. Table 3 summarizes the differences between the six radioactive agents that have been investigated as a brachytherapy in the management of pancreatic cancer.

**Table 3:** Differences between the 5 radio-isotopes used for brachytherapy in pancreatic cancer.

Radioisotope	Half-Life	Radiation Type and Penetration	Typical Dose	Delivery Method	Clinical Stage/Use	Advantages	Limitations
**^125^I [49,50,51,52,53,54,55]**	60 days	γ-rays, moderate penetration (~1–2 cm)	140–160 Gy	EUS-guided seed implantation	Unresectable PDAC (Stage III–IV)	Long half-life allows sustained radiation	Multiple punctures required; stiff 19G needle; limited survival benefit; risk of pancreatitis
**^32^P [56,57,58,59,60,61,62]**	16 days	β-particles, short penetration (<1 cm)	100–400 Gy	EUS-guided microparticles	LAPC, metastatic PDAC	High localized dose; minimally invasive; synergistic with concurrent chemo; enhances vascularity	Requires imaging to confirm distribution; limited long-term data
**^166^HoMS [63,64]**	26.8 h	β^−^, short penetration (~1 cm)	Equivalent high dose rate	Intratumoral injection	Unresectable PDAC (experimental)	High dose rate; multimodality imaging (SPECT/CT/MRI); steep dose fall-off	Very limited human data; efficacy unproven
**^103^Pd [65,66]**	17 days	Low-energy photons, rapid dose fall-off with penetration of 1.7 cm	~100–115 Gy	Intraoperative interstitial implant	Unresectable PDAC	Favorable dose distribution; palliation of pain	Small studies; no survival benefit; risk of grade ≥ 3 complications
**^90^Y [67,68,69,70,71]**	64.5 h	β-particles, moderate penetration (~5–10 mm)	Variable, high	TARE or EUS-guided intratumoral	Liver metastases from PDAC; unresectable PDAC (experimental)	High local dose; rapid energy delivery; potential for image-guided placement	Mostly preclinical or hepatic metastases; clinical efficacy in pancreatic primary under investigation
**^192^Ir [72]**	73.8 days	γ-rays (5–10 mm)	20 Gy	Intratumoral injection (intraoperative)	Unresectable PDAC/pain relief	Shown to improve pain and quality of life	Requires surgical catheter placement

EUS = endoscopic ultrasound; PDAC = pancreatic ductal adenocarcinoma; LAPC = locally advanced pancreatic cancer; ^125^I = iodine-125; ^32^P = phosphorus-32; ^166^HoMS = Holmium-166 Poly-L-Lactic Acid Microspheres; ^103^Pd = palladium-103; ^90^Y = yttrium-90; ^192^Ir = iridium 192; TARE = transarterial radioembolization; SPECT = single-photon-emission computed tomography; CT = computed tomography; MRI = magnetic resonance imaging.

## 6. Conclusions

The therapeutic role of EUS-guided fine-needle injection is rapidly expanding and represents a significant advancement in the management of pancreatic ductal adenocarcinoma (PDAC), particularly for patients with locally advanced or metastatic disease who are ineligible for surgical resection. The options are vast, with some therapies demonstrating more obvious benefit. Although EUS-guided RFA, chemotherapy, immune and gene therapy have not shown an overwhelming response in PDAC, the advent of EUS-guided intra-tumoral radioactive implantation has revealed exciting results in its early studies. Among the available options, EUS-guided phosphorus-32 (^32^P) microparticle brachytherapy has shown promising results, including substantial tumor shrinkage, reductions in CA 19-9 levels, and increased rates of tumor downstaging and surgical conversion when combined with systemic chemotherapy. Compared with stereotactic body radiotherapy (SBRT), ^32^P offers several distinct advantages: it delivers higher intratumoral radiation doses, spares surrounding healthy tissue, and can be administered concurrently with ongoing chemotherapy without interrupting systemic treatment. Emerging evidence also indicates that ^32^P may modulate the tumor microenvironment, improving vascularity and enhancing the efficacy of chemotherapy. The procedure has demonstrated high technical success rates and a favorable safety profile, with few serious adverse events and only minor, manageable complications.

Future research should prioritize prospective, randomized controlled trials to confirm its effects on local tumor control and overall survival, while refining optimal dosing strategies and treatment protocols. EUS-guided ^32^P brachytherapy has the potential to become a key component of multimodal therapy, bridging local tumor control with systemic disease management in PDAC. Concurrently, continued investigation into alternative radioactive isotopes and other intra-tumoral therapeutic modalities remains essential to further expand treatment options and improve outcomes.

## Figures and Tables

**Table 1:** Prospective clinical studies of EUS-guided intra-tumor therapies in locally advanced pancreatic cancer.

Modalities	Types of Study	Patient Group	Intra-Tumor Therapy	Additional Therapy	Response Outcome	Median Survival (Months)
**Chemotherapy**						
Levy M et al. (2017) [17]	Prospective	Stage II, III, IV	Gemcitabine	5-FU with or without RT	PR 25% SD 57% Downstaging with resection 20% (4/20 patients of stage III disease)	10.4
**Radiofrequency ablation (RFA)**						
Song et al. (2016) [18]	Prospective	LAPC and mPDAC	RFA 20–50 W	50% with Gemcitabine	Technically feasible and safe	NR
Crino SF et al. (2018) [19]	Prospective	LAPC	RFA 30 W	Standard CTX with or without RT	Mean volume reduction 30% of tumor mass	NR
Scopelliti F et al. (2018) [22]	Prospective	LAPC	RFA 30 W (> 3 cm) or 20 W (<3 cm)	Standard CTX with or without RT	Tumor size reduction 50%	NR
Thosani N et al. (2022) [23]	Prospective	LAPC and mPDAC	RFA 15–20 W up to 4 sessions	Standard CTX	7/10 patients with tumor progression 1/10 downstaging with R0 resection	13.4
Oh D et al. (2022) [21]	Prospective	LAPC and mPDAC	RFA 50 W	Standard CTX	Local progression free 6.83 months	24.03
Tiankanon K et al. (2019) [24]	Prospective, open-labeled	LAPC	RFA 50 W	Standard CTX with RFA vs. CTX alone	No significant tumor size in median maximal tumor diameter between both groups	Survival rate 70% at 6 months
Kongkam P et al. (2025) [20]	Prospective, propensity score matched	LAPC	RFA 50 W	Standard CTX with RFA vs. CTX alone	Hazard ratio 0.5, *p* = 0.03 Survival probability at 12 months 58% in EUS-RFA compared to 22% for controls	13.4
**Immune-induced local therapy**						
Chang KJ et al. (2000) [26]	Prospective	Stage II, III, IV	Mixed lymphocyte culture	None	PR 25% Minor response 1%	13.2
Irisawa A et al. (2007) [33]	Prospective	Stage IV	Immature DCs	RT	SD 26% Minor response 26%	9.9
Hirooka Y et al. (2009) [30]	Prospective	Stage III	OK-432 primed immature DCs	Gemcitabine, CD3-LAK antibodies	SD 40% PR 20%	15.9
Hirooka Y et al. (2017) [32]	Prospective	LAPC	Zolendronate-pulsed immature DCs	Gemcitabine, αβ T cells	SD 40%	11.5
**Gene-induced local therapy**						
Hecht JR et al. (2003) [27]	Prospective	Stage III and IV	ONYX-15 (adenovirus)	Gemcitabine	PR 9.5% (2/21) Minor response 9.5% (2/21) SD 28.6% (6/11)	7.5
Hecht JR et al. (2012) [28]	Prospective	LAPC	TNFerade	5-FU, external RT	CR 2% (1/50) PR 6% (3/50) SD 34% (12/50)	9.9
Herman JM et al. (2013) [29]	RCT	LAPC	TNFerade	5-FU, RT, gemcitabine ± erlotinib	No difference in local control	10
Hirooka Y et al. (2018) [31]	Single arm, open-label Phase I trial	Stage III	HF10 Oncolytic virus	Erlotinib and gemcitabine	PR 30% SD40% PD 20% Downstaging with resection 20%	15.5

CR = complete response; CTX = chemotherapy; DCs = dendritic cells; LAPC = locally advanced pancreatic cancer; mPDAC = metastatic pancreatic adenocarcinoma; PD = progressive disease; PR = partial response; RFA = radiofrequency ablation; RCT = randomized controlled trial; RT = radiotherapy; SD = stable disease.

**Table 2:** The differences between SBRT and ^32^P brachytherapy in PDAC [41,42,43,44,45,54,55,56,57,58,59,60,61,62].

Feature	SBRT (Stereotactic Body Radiotherapy)	^32^P EUS-Guided Brachytherapy
**Timing with Chemotherapy**	Usually delivered after completion of systemic chemotherapy	Can be implanted early, during initial chemotherapy cycles, allowing concurrent therapy
**Radiation Dose**	Typically 40–50 Gy (external beam)	Higher localized dose: 100–400 Gy directly to tumor
**Radiation Delivery**	External beam, penetrates surrounding tissues	Beta radiation with short penetration (<1 cm), minimizing exposure to adjacent organs
**Procedure**	Requires fiducial markers, CT planning, and multiple sessions (3–5 fractions)	Single-session EUS-guided implantation using microparticles
**Treatment Delay**	Planning and fiducial placement can delay restaging or surgery	Minimal delay; systemic chemotherapy can resume soon after implantation
**Tumor Microenvironment**	May induce immunogenic cell death but limited effect on vascularity or stromal barrier	May enhance microvascular flow and overcome dense stroma, improving chemotherapy delivery
**Surgical Conversion Potential**	Limited; performed after chemotherapy	Higher downstaging and resection rates observed when combined with chemotherapy
**Monitoring**	Imaging-based assessment post-treatment	Bremsstrahlung imaging or SPECT-CT immediately after implantation for precise localization
**Side Effects**	Risk of radiation-induced injury to adjacent organs; grade 3–4 toxicity up to 37.7%	Generally safe; minor self-limited events, serious procedure-related complications rare (<10%)
**Local Control**	Good but limited by dose constraints	High local control rates reported (up to 100% at 3 months in metastatic PDAC)

SBRT = stereotactic body radiation therapy; ^32^P = phosphorus-32; PDAC = pancreatic ductal adenocarcinoma; EUS = endoscopic ultrasound; SPECT-CT = single-photon-emission computed tomography.

## Data Availability

This review is inspired by the recent published data by the authors on the topic of intra-tumorual radiative isotope implantation for pancreatic cancer. Not only the additional use of ^32^P to standard chemotherapy has survival benefit over chemotherapy alone, the combination therapy has also been shown to alter the tumor microenvironment, namely vascularity. This work is published in Cancers in 2024.

## References

[1] Bray F., Laversanne M., Sung H., Ferlay J., Siegel R.L., Soerjomataram I., Jemal A. (2024). Global cancer statistics 2022: GLOBOCAN estimates of incidence and mortality worldwide for 36 cancers in 185 countries. CA Cancer J. Clin..

[2] Siegel R.L., Kratzer T.B., Giaquinto A.N., Sung H., Jemal A. (2025). Cancer statistics, 2025. CA Cancer J. Clin..

[3] Rawla P., Sunkara T., Gaduputi V. (2019). Epidemiology of Pancreatic Cancer: Global Trends, Etiology and Risk Factors. World J. Oncol..

[4] Conroy T., Pfeiffer P., Vilgrain V., Lamarca A., Seufferlein T., O’reilly E., Hackert T., Golan T., Prager G., Haustermans K. (2023). Pancreatic cancer: ESMO Clinical Practice Guideline for diagnosis, treatment and follow-up. Ann. Oncol..

[5] Balaban E.P., Mangu P.B., Khorana A.A., Shah M.A., Mukherjee S., Crane C.H., Javle M.M., Eads J.R., Allen P., Ko A.H. (2016). Locally Advanced, Unresectable Pancreatic Cancer: American Society of Clinical Oncology Clinical Practice Guideline. J. Clin. Oncol..

[6] Tempero M.A., Malafa M.P., Al-Hawary M., Behrman S.W., Benson A.B., Cardin D.B., Chiorean E.G., Chung V., Czito B., Del Chiaro M. (2021). Pancreatic Adenocarcinoma, Version 2.2021, NCCN Clinical Practice Guidelines in Oncology. J. Natl. Compr. Cancer Netw..

[7] Jiang Y., Sohal D.P. (2023). Pancreatic Adenocarcinoma Management. JCO Oncol. Pract..

[8] Quiñonero F., Mesas C., Doello K., Cabeza L., Perazzoli G., Jimenez-Luna C., Rama A.R., Melguizo C., Prados J. (2019). The challenge of drug resistance in pancreatic ductal adenocarcinoma: A current overview. Cancer Biol. Med..

[9] Anderson N.M., Simon M.C. (2020). The tumor microenvironment. Curr. Biol..

[10] Erkan M., Adler G., Apte M.V., Bachem M.G., Buchholz M., Detlefsen S., Esposito I., Friess H., Gress T.M., Habisch H.J. (2012). StellaTUM: Current consensus and discussion on pancreatic stellate cell research. Gut.

[11] Vaish U., Jain T., Are A.C., Dudeja V. (2021). Cancer-Associated Fibroblasts in Pancreatic Ductal Adenocarcinoma: An Update on Heterogeneity and Therapeutic Targeting. Int. J. Mol. Sci..

[12] De Grandis M.C., Ascenti V., Lanza C., Di Paolo G., Galassi B., Ierardi A.M., Carrafiello G., Facciorusso A., Ghidini M. (2023). Locoregional Therapies and Remodeling of Tumor Microenvironment in Pancreatic Cancer. Int. J. Mol. Sci..

[13] Ramanathan R.K., McDonough S.L., Philip P.A., Hingorani S.R., Lacy J., Kortmansky J.S., Thumar J., Chiorean E.G., Shields A.F., Behl D. (2019). Phase IB/II Randomized Study of FOLFIRINOX Plus Pegylated Recombinant Human Hyaluronidase Versus FOLFIRINOX Alone in Patients With Metastatic Pancreatic Adenocarcinoma: SWOG S1313. J. Clin. Oncol..

[14] Van Cutsem E., Tempero M.A., Sigal D., Oh D.-Y., Fazio N., Macarulla T., Hitre E., Hammel P., Hendifar A.E., Bates S.E. (2020). Randomized Phase III Trial of Pegvorhyaluronidase Alfa With Nab-Paclitaxel Plus Gemcitabine for Patients With Hyaluronan-High Metastatic Pancreatic Adenocarcinoma. J. Clin. Oncol..

[15] Mulvihill S., Warren R., Venook A., Adler A., Randlev B., Heise C., Kirn D. (2001). Safety and feasibility of injection with an E1B-55 kDa gene-deleted, replication-selective adenovirus (ONYX-015) into primary carcinomas of the pancreas: A phase I trial. Gene Ther..

[16] Sooklal S., Chahal P. (2020). Endoscopic Ultrasound. Surg. Clin. N. Am..

[17] Levy M.J., Alberts S.R., Bamlet W.R., Burch P.A., Farnell M.B., Gleeson F.C., Haddock M.G., Kendrick M.L., Oberg A.L., Petersen G.M. (2017). EUS-guided fine-needle injection of gemcitabine for locally advanced and metastatic pancreatic cancer. Gastrointest. Endosc..

[18] Song T.J., Seo D.W., Lakhtakia S., Reddy N., Oh D.W., Park D.H., Lee S.S., Lee S.K., Kim M.-H. (2016). Initial experience of EUS-guided radiofrequency ablation of unresectable pancreatic cancer. Gastrointest. Endosc..

[19] Crinò S.F., D’oNofrio M., Bernardoni L., Frulloni L., Iannelli M., Malleo G., Paiella S., Larghi A., Gabbrielli A. (2018). EUS-guided Radiofrequency Ablation (EUS-RFA) of Solid Pancreatic Neoplasm Using an 18-gauge Needle Electrode: Feasibility, Safety, and Technical Success. J. Gastrointest. Liver Dis..

[20] Kongkam P., Tantitanawat K., Kerr S., Lopimpisuth C., Tiankanon K., Angsuwatcharakon P., Ridtitid W., Mekaroonkamol P., Teeyapun N., Tanasanvimon S. (2025). One-year survival rate of unresectable pancreatic cancer size 4 cm or smaller treated with or without EUS-radiofrequency ablation. Gastrointest. Endosc..

[21] Oh D., Seo D.-W., Song T.J., Park D.H., Lee S.K., Kim M.-H. (2022). Clinical outcomes of EUS-guided radiofrequency ablation for unresectable pancreatic cancer. Endosc. Ultrasound.

[22] Scopelliti F., Pea A., Conigliaro R., Butturini G., Frigerio I., Regi P., Giardino A., Bertani H., Paini M., Pederzoli P. (2018). Technique, safety, and feasibility of EUS-guided radiofrequency ablation in unresectable pancreatic cancer. Surg. Endosc..

[23] Thosani N., Cen P., Rowe J., Guha S., Bailey-Lundberg J.M., Bhakta D., Patil P., Wray C.J. (2022). Endoscopic ultrasound-guided radiofrequency ablation (EUS-RFA) for advanced pancreatic and periampullary adenocarcinoma. Sci. Rep..

[24] Tiankanon K., Kongkam P., Orprayoon T., Luangsukrerk T., Seo D., Sriuranpong V., Nantavithya C., Jantarattana T., Cañones A., Angsuwatcharakon P. (2019). EUS-guided radiofrequency ablation plus chemotherapy versus chemotherapy alone for unresectable pancreatic cancer (ERAP): Preliminary results of a prospective comparative study. Endoscopy.

[25] Khoury T., Sbeit W., Napoléon B. (2023). Endoscopic ultrasound guided radiofrequency ablation for pancreatic tumors: A critical review focusing on safety, efficacy and controversies. World J. Gastroenterol..

[26] Chang K.J., Nguyen P.T., Thompson J.A., Kurosaki T.T., Casey L.R., Leung E.C., Granger G.A. (2000). Phase I clinical trial of allogeneic mixed lymphocyte culture (cytoimplant) delivered by endoscopic ultrasound?guided fine-needle injection in patients with advanced pancreatic carcinoma. Cancer.

[27] Hecht J.R., Bedford R., Abbruzzese J.L., Lahoti S., Reid T.R., Soetikno R.M., Kirn D.H., Freeman S.M. (2003). A phase I/II trial of intratumoral endoscopic ultrasound injection of ONYX-015 with intravenous gemcitabine in unresectable pancreatic carcinoma. Clin. Cancer Res..

[28] Hecht J.R., Farrell J.J., Senzer N., Nemunaitis J., Rosemurgy A., Chung T., Hanna N., Chang K.J., Javle M., Posner M. (2012). EUS or percutaneously guided intratumoral TNFerade biologic with 5-fluorouracil and radiotherapy for first-line treatment of locally advanced pancreatic cancer: A phase I/II study. Gastrointest. Endosc..

[29] Herman J.M., Wild A.T., Wang H., Tran P.T., Chang K.J., Taylor G.E., Donehower R.C., Pawlik T.M., Ziegler M.A., Cai H. (2013). Randomized Phase III Multi-Institutional Study of TNFerade Biologic With Fluorouracil and Radiotherapy for Locally Advanced Pancreatic Cancer: Final Results. J. Clin. Oncol..

[30] Hirooka Y., Itoh A., Kawashima H., Hara K., Nonogaki K., Kasugai T., Ohno E., Ishikawa T., Matsubara H., Ishigami M. (2009). A Combination Therapy of Gemcitabine With Immunotherapy for Patients With Inoperable Locally Advanced Pancreatic Cancer. Pancreas.

[31] Hirooka Y., Kasuya H., Ishikawa T., Kawashima H., Ohno E., Villalobos I.B., Naoe Y., Ichinose T., Koyama N., Tanaka M. (2018). A Phase I clinical trial of EUS-guided intratumoral injection of the oncolytic virus, HF10 for unresectable locally advanced pancreatic cancer. BMC Cancer.

[32] Hirooka Y., Kawashima H., Ohno E., Ishikawa T., Kamigaki T., Goto S., Takahara M., Goto H. (2017). Comprehensive immunotherapy combined with intratumoral injection of zoledronate-pulsed dendritic cells, intravenous adoptive activated T lymphocyte and gemcitabine in unresectable locally advanced pancreatic carcinoma: A phase I/II trial. Oncotarget.

[33] Irisawa A., Takagi T., Kanazawa M., Ogata T., Sato Y., Takenoshita S.-I., Ohto H., Ohira H. (2007). Endoscopic Ultrasound-Guided Fine-Needle Injection of Immature Dendritic Cells Into Advanced Pancreatic Cancer Refractory to Gemcitabine. Pancreas.

[34] Xu Y., Chen J., Qiu Y., Du J. (2025). Radiotherapy and immune microenvironment crosstalk in pancreatic cancer: A comprehensive review of current insights and future directions. Front. Immunol..

[35] Costa A.D., Väyrynen S.A., Chawla A., Zhang J., Väyrynen J.P., Lau M.C., Williams H.L., Yuan C., Morales-Oyarvide V., Elganainy D. (2022). Neoadjuvant Chemotherapy Is Associated with Altered Immune Cell Infiltration and an Anti-Tumorigenic Microenvironment in Resected Pancreatic Cancer. Clin. Cancer Res..

[36] Salamekh S., Gottumukkala S., Park C., Lin M.-H., Sanford N.N. (2022). Radiotherapy for Pancreatic Adenocarcinoma. Hematol. Clin. N. Am..

[37] Yasuda S., Nagai M., Nakamura K., Matsuo Y., Sho M. (2025). Role of radiotherapy in surgical approaches to pancreatic cancer treatment: A narrative review. Ann. Gastroenterol. Surg..

[38] Malla M., Fekrmandi F., Malik N., Hatoum H., George S., Goldberg R.M., Mukherjee S. (2023). The evolving role of radiation in pancreatic cancer. Front. Oncol..

[39] Ermongkonchai T., Khor R., Muralidharan V., Tebbutt N., Lim K., Kutaiba N., Ng S.P. (2022). Stereotactic radiotherapy and the potential role of magnetic resonance-guided adaptive techniques for pancreatic cancer. World J. Gastroenterol..

[40] Ejlsmark M.W., Bahij R., Schytte T., Hansen C.R., Bertelsen A., Mahmood F., Mortensen M.B., Detlefsen S., Weber B., Bernchou U. (2024). Adaptive MRI-guided stereotactic body radiation therapy for locally advanced pancreatic cancer—A phase II study. Radiother. Oncol..

[41] Tchelebi L.T., Lehrer E.J., Trifiletti D.M., Sharma N.K., Gusani N.J., Crane C.H., Zaorsky N.G. (2020). Conventionally fractionated radiation therapy versus stereotactic body radiation therapy for locally advanced pancreatic cancer (CRiSP): An international systematic review and meta-analysis. Cancer.

[42] Cazacu I.M., Singh B.S., Martin-Paulpeter R.M., Beddar S., Chun S., Holliday E.B., Koong A.C., Das P., Koay E.J., Taniguchi C. (2023). Endoscopic Ultrasound-Guided Fiducial Placement for Stereotactic Body Radiation Therapy in Patients with Pancreatic Cancer. Cancers.

[43] Kerdsirichairat T., Shin E.J. (2020). Role of endoscopic ultrasonography guided fiducial marker placement in gastrointestinal cancer. Curr. Opin. Gastroenterol..

[44] Sanders M.K., Moser A.J., Khalid A., Fasanella K.E., Zeh H.J., Burton S., McGrath K. (2010). EUS-guided fiducial placement for stereotactic body radiotherapy in locally advanced and recurrent pancreatic cancer. Gastrointest. Endosc..

[45] Mills B.N., Qiu H., Drage M.G., Chen C., Mathew J.S., Garrett-Larsen J., Ye J., Uccello T.P., Murphy J.D., Belt B.A. (2021). Modulation of the Human Pancreatic Ductal Adenocarcinoma Immune Microenvironment by Stereotactic Body Radiotherapy. Clin. Cancer Res..

[46] DeNittis A.S., Stambaugh M.D., Lang P., Wallner P.E., Lustig R.A., Dillman R.O., Order S.E. (1999). Complete Remission of Nonresectable Pancreatic Cancer After Infusional Colloidal Phosphorus-32 Brachytherapy, External Beam Radiation Therapy, and 5-Fluorouracil. Am. J. Clin. Oncol..

[47] Han J., Chang K.J. (2017). Endoscopic Ultrasound-Guided Direct Intervention for Solid Pancreatic Tumors. Clin. Endosc..

[48] Larghi A., Rimbaș M., Rizzatti G., Carbone C., Gasbarrini A., Costamagna G., Alfieri S., Tortora G. (2021). Endoscopic ultrasound-guided therapies for pancreatic solid tumors: An overview. Semin. Oncol..

[49] Wei S., Li C., Li M., Xiong Y., Jiang Y., Sun H., Qiu B., Lin C.J., Wang J. (2021). Radioactive Iodine-125 in Tumor Therapy: Advances and Future Directions. Front. Oncol..

[50] Jin Z., Du Y., Li Z., Jiang Y., Chen J., Liu Y. (2008). Endoscopic ultrasonography-guided interstitial implantation of iodine 125-seeds combined with chemotherapy in the treatment of unresectable pancreatic carcinoma: A prospective pilot study. Endoscopy.

[51] Sun S., Xu H., Xin J., Liu J., Guo Q., Li S. (2006). Endoscopic Ultrasound-Guided Interstitial Brachytherapy of Unresectable Pancreatic Cancer: Results of a Pilot Trial. Endoscopy.

[52] Sun X., Lu Z., Wu Y., Min M., Bi Y., Shen W., Xu Y., Li Z., Jin Z., Liu Y. (2017). An endoscopic ultrasonography-guided interstitial brachytherapy based special treatment-planning system for unresectable pancreatic cancer. Oncotarget.

[53] Du Y.-Q., Jin Z., Meng H., Zou D., Chen J., Liu Y., Zhan X., Wang D., Liao Z., Li Z. (2013). Long-term effect of gemcitabine-combined endoscopic ultrasonography-guided brachytherapy in pancreatic cancer. J Interv Gastroenterol..

[54] Han Q., Deng M., Lv Y., Dai G. (2017). Survival of patients with advanced pancreatic cancer after iodine125 seeds implantation brachytherapy. Medicine.

[55] Lim A.H., Sukocheva O., Bednarz J., Bartholomeusz D., Rayner C.K., Tse E., Nguyen N.Q. (2025). Impact of Different Modalities of Radiotherapy in Locally Advanced Pancreatic Cancer: A Review and Meta-Analysis. J. Gastroenterol. Hepatol..

[56] Gholami Y.H., Wilson N., James D., Kuncic Z. (2017). Toward Personalized Dosimetry with 32 P Microparticle Therapy for Advanced Pancreatic Cancer. Int. J. Radiat. Oncol..

[57] Ross P., Wasan H., Croagh D., Nikfarjam M., Nguyen N., Aghmesheh M., Nagrial A., Bartholomeusz D., Hendlisz A., Ajithkumar T. (2021). Results of a single-arm pilot study of 32P microparticles in unresectable locally advanced pancreatic adenocarcinoma with gemcitabine/nab-paclitaxel or FOLFIRINOX chemotherapy. ESMO Open.

[58] Lim A.H., Nitchingham D., Bednarz J., Bills M., Lanka L., Allen B., Tan A., Joshi R., Hsieh W., Crouch B. (2025). Combined phosphorus-32 implantation and chemotherapy versus chemotherapy alone for locally advanced pancreatic cancer: A propensity score–weighted landmark analysis. Gastrointest. Endosc..

[59] Lim A.H., Ali F., Zobel J., Safaeian R., Phan V.-A., Singhal N., Bartholomeusz D., Jin A., Rayner C.K., Tse E. (2025). Tu1372: Comparison of combined chemotherapy and stereotactic body radiation therapy with combined chemotherapy and phosphorus-32 microparticle intra-tumoural implantation in patients with locally advanced pancreatic adenocarcinoma. Gastroenterology.

[60] Lim A.H.W., Zobel J., Bills M., Hsieh W., Crouch B., Joshi R., Thomson J.-E., Neo E., Kuan L.L., Safaeian R. (2024). The Impact of Combined Chemotherapy and Intra-Tumoural Injection of Phosphorus-32 Microparticles on Vascularity in Locally Advanced Pancreatic Carcinoma. Cancers.

[61] Naidu J., Bartholomeusz D., Zobel J., Safaeian R., Hsieh W., Crouch B., Ho K., Calnan D., Singhal N., Ruszkiewicz A. (2021). Combined chemotherapy and endoscopic ultrasound-guided intratumoral 32P implantation for locally advanced pancreatic adenocarcinoma: A pilot study. Endoscopy.

[62] Lim A.H.W., Singhal N., Bartholomeusz D., Zobel J., Naidu J., Hsieh W., Crouch B., Wasan H., Croagh D., Nagrial A. (2024). Outcomes of phosphorus-32 microparticle intratumoral implantation added to chemotherapy in patients with metastatic pancreatic adenocarcinoma. iGIE.

[63] Klaassen N.J.M., Arntz M.J., Gil Arranja A., Roosen J., Nijsen J.F.W. (2019). The various therapeutic applications of the medical isotope holmium-166: A narrative review. EJNMMI Radiopharm. Chem..

[64] Willink C.Y., Jenniskens S.F., Stommel M.W., Janssen M.J., Hermans J.J., Westdorp H., van Laarhoven C.J., Fütterer J.J., Nijsen J.F.W. (2025). Intratumoral Holmium-166 Microsphere Injection in Patients with Unresectable Pancreatic Ductal Adenocarcinoma: A Single-Center, Single-Arm, Open-Label Feasibility and Safety Study. Dig. Surg..

[65] Nori D., Merimsky O., Osian A.D., Heffernan M., Cortes E., Turner J.W. (1996). Palladium-103: A new radioactive source in the treatment of unresectable carcinoma of the pancreas: A phase I–II study. J. Surg. Oncol..

[66] Raben A., Mychalczak B., Brennan M.F., Minsky B., Anderson L., Casper E.S., Harrison L.B. (1996). Feasibility study of the treatment of primary unresectable carcinoma of the pancreas with 103PD brachytherapy. Int. J. Radiat. Oncol..

[67] Salem R., Gordon A.C., Mouli S., Hickey R., Kallini J., Gabr A., Mulcahy M.F., Baker T., Abecassis M., Miller F.H. (2016). Y90 Radioembolization Significantly Prolongs Time to Progression Compared With Chemoembolization in Patients With Hepatocellular Carcinoma. Gastroenterology.

[68] Kayaleh R., Krzyston H., Rishi A., Naziri J., Frakes J., Choi J., El-Haddad G., Parikh N., Sweeney J., Kis B. (2020). Transarterial Radioembolization Treatment of Pancreatic Cancer Patients with Liver-Dominant Metastatic Disease Using Yttrium-90 Glass Microspheres: A Single-Institution Retrospective Study. J. Vasc. Interv. Radiol..

[69] Kim A.Y., Frantz S., Brower J., Akhter N. (2019). Radioembolization with Yttrium-90 Microspheres for the Treatment of Liver Metastases of Pancreatic Adenocarcinoma: A Multicenter Analysis. J. Vasc. Interv. Radiol..

[70] Michl M., Haug A., Jakobs T., Paprottka P., Hoffmann R.-T., Bartenstein P., Boeck S., Haas M., Laubender R., Heinemann V. (2013). Radioembolization with Yttrium-90 Microspheres (SIRT) in Pancreatic Cancer Patients with Liver Metastases: Efficacy, Safety and Prognostic Factors. Oncology.

[71] Zhao Y., Yang Y., Zhang B., Cui H., Liu L., Wang R., Han Y., Zhu D., Ma W., Zhang X. (2025). Endoscopic Ultrasound-Guided Brachytherapy of Yttrium-90 Implantation Into Pancreas: A Dose-Escalation Pilot Study. Medcomm.

[72] Waniczek D., Piecuch J., Rudzki M., Mikusek W., Arendt J., Białas B. (2011). Clinical Investigations Perioperative high dose rate (HDR) brachytherapy in unresectable locally advanced pancreatic tumors. J. Contemp. Brachytherapy.

